# Entrectinib-related Myocarditis Causing a Triangular QRS-ST-T Waveform Electrocardiographic Pattern: A Case Report

**DOI:** 10.5811/cpcem.53200

**Published:** 2026-08-04

**Authors:** William Dean, James Dean, Hady Lichaa, Christopher Wilbert

**Affiliations:** University of Tennessee Health Science Center, Ascension Saint Thomas Rutherford Hospital, Emergency Medicine Residency Program, Murfreesboro, Tennessee

**Keywords:** entrectinib, myocarditis, triangular QRS-ST-T wave, shark-fin ECG, case report

## Abstract

**Introduction:**

Entrectinib is a kinase inhibitor used in ROS1-positive non-small cell lung carcinoma. Cardiovascular toxicity is rare, with only one prior report of myocarditis related to entrectinib use. The triangular QRS-ST-T waveform electrocardiographic pattern is a ST-elevated myocardial infarction equivalent and is rarely associated with myocarditis.

**Case Report:**

A 42-year-old male with metastatic non-small cell lung carcinoma presented with presyncope, palpitations, and dyspnea three days after initiating entrectinib. Electrocardiograph (ECG) revealed a “shark-fin” T-wave morphology with diffuse ST elevation, QTc prolongation, and elevated troponin. ST-elevated myocardial infarction protocol was initiated with tenecteplase, heparin, clopidogrel, and aspirin. Left heart catheterization was normal. The ECG showed an ejection fraction of 20–25% with global hypokinesis. Myocarditis with heart failure associated with entrectinib use was suspected. Cardiac magnetic resonance imaging confirmed myopericarditis with subsequent recovery of ejection fraction to 57%. The patient was discharged after five days with complete recovery.

**Conclusion:**

This is the second reported case of myocarditis associated with entrectinib use and the second documented case of myocarditis presenting with a T-wave ECG pattern. Clinicians should be aware that entrectinib is associated with early-onset myocarditis and that the T-wave pattern, while strongly correlated to occlusive myocardial infarction, may rarely occur in myocarditis. Prompt recognition is critical to avoid mismanagement.

## INTRODUCTION

A 42-year-old male who presented to the emergency department (ED) with an electrocardiograph (ECG) concerning for ST-elevated myocardial infarction (STEMI) was found to have myocarditis with heart failure associated with entrectinib use. On literature review, we found only two other documented cases of entrectinib-related cardiovascular events; this is the second case of myocarditis.^1^,^2^ This is also the second reported case of myocarditis causing a triangular QRS-ST-T waveform ECG pattern.^3^

## CASE REPORT

**Image 1:**
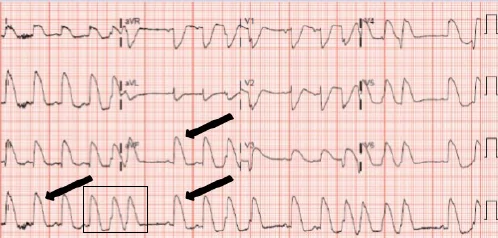
Electrocardiograph with a shark-fin ST-elevation morphology known as triangular QRS-ST-T waveform diffusely with QTc prolongation. Black arrows indicate classic shark-fin waveform. Also note short episode of non-sustained ventricular tachycardia, denoted by black box.

**Image 2:**
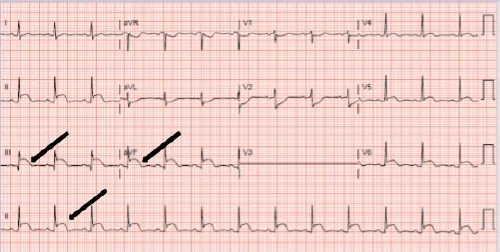
Post-admission electrocardiograph (ECG) demonstrating classical ST elevation and ischemic changes. Black arrows indicate ST elevation seen in post-admission ECG.

Three days after starting entrectinib, a 42-year-old male with non-small cell lung carcinoma presented with presyncope, palpitations, and dyspnea. In the ED, the patient had an ECG with a “shark-fin” ST-elevation morphology known as T waveform diffusely with QTc prolongation concerning for occlusive myocardial infarction (Image 1). Cardiology consult recommended tenecteplase, heparin, clopidogrel, and aspirin. The ECG remained unchanged, but the patient did not decompensate. After admission, the ECG progressed to classic ST-elevation and eventual ischemic findings (Image 2).

An echocardiogram showed severely reduced systolic function with left ventricular ejection fraction of 20–25% and global hypokinesis without pericardial effusion. Serial troponins maintained a relatively flat trend; the highest troponin I obtained was 0.19 nanograms per milliliter (ng/mL) (reference range: <0.04 ng/mL) on arrival to the ED. Repeat troponin I levels were 0.17 and 0.14 ng/mL. Coronary angiography demonstrated normal flow and, combined with troponin I findings, supported a non-occlusive etiology of myocardial injury. Entrectinib-related myocarditis and acute heart failure was suspected. Cardiac magnetic resonance imaging later showed improved left ventricular ejection fraction of 57% and myopericarditis, confirming the diagnosis. The patient was discharged five days later after supportive care and has since made a complete recovery.

*CPC-EM Capsule*

What do we already know about this clinical entity?

*Entrectinib is a kinase inhibitor used in metastatic non-small cell lung carcinoma. Cardiotoxicity is rare.*

What makes this presentation of disease reportable?

*The patient’s entrectinib-associated myocarditis presented with a triangular shark-fin QRS-ST-T ECG pattern, a* ST-elevated myocardial infarction (*STEMI) equivalent rarely reported in myocarditis.*

What is the major learning point?

*The QRS-ST-T wave suggests occlusive myocardial infarction but can rarely occur in myocarditis, including cardiotoxicity from targeted cancer therapies.*

How might this improve emergency medicine practice?

*Clinicians should consider myocarditis and cardiotoxic medications in STEMI mimics, especially in oncology patients on kinase inhibitors.*

## DISCUSSION

Entrectinib is a kinase inhibitor under accelerated U.S. Food and Drug Administration approval for non-surgical ROS1-positive metastatic non-small cell lung carcinoma. During clinical trials, congestive heart failure occurred in 3.4% of patients within two months, while only one case of myocarditis was reported, which is not currently listed as a potential side effect.^4^ Molecularly targeted therapies for metastatic non-small cell lung carcinoma have overtaken cytotoxic chemotherapy as first-line treatment for patients with actionable genetic deviations.^5^ Although generally better tolerated, cardiovascular-adverse drug reactions have been documented, most commonly congestive heart failure, arrhythmias, and QTc prolongation, while myocarditis remains underreported.^6^

Notably, the time course of cardiotoxicity can be highly variable, with reports of congestive heart failure or myocarditis emerging as early as two weeks after drug initiation or as late as one month despite treatment interruption. This unpredictable latency highlights the need for close monitoring in the initial weeks of therapy; it also emphasizes the need to consider myocarditis in patients on entrectinib regardless of the initial timeline of medication administration.^7^,^8^ The nature of the relationship between entrectinib and myocarditis is most accurately characterized as an association rather than causation. However, with increasing numbers of reports of myocarditis associated with entrectinib, further prospective studies would be beneficial to elucidate any causality in these findings.

The ECG finding known as the “shark-fin” pattern, or T wave, has been shown to represent an occlusive myocardial infarction (and is typically managed as a STEMI equivalent). Occlusive myocardial infarctions with this pattern carry increased risk of ventricular fibrillation and high mortality.^9^ This pattern has also been mistaken on initital review for ventricular tachycardia, contributing to potentially dangerous treatment errors.^10^ Importantly, although this finding is almost exclusively associated with occlusive myocardial infarction, it has also been documented in rare myocarditis cases, including one fulminant entrectinib-induced myocarditis where emergent coronary angiography revealed no obstructive disease.^7^ This overlap highlights the diagnostic challenge and potential for mismanagement when myocarditis presents with misleading ECG characteristics.

When myocarditis is suspected, management strategies extend beyond simple discontinuation of the agent. While our patient improved with supportive care, other case reports describe the use of high-dose corticosteroids and advanced mechanical support such as intraaortic balloon pump and extracorporeal membrane oxygenation to stabilize fulminant presentations.^8^,^11^ These findings underscore the importance of rapid recognition, multidisciplinary care, and tailoring interventions based on severity, as misclassification can rapidly lead to suboptimal care and poor recognition of progressing cardiotoxicity.

## CONCLUSION

This case demonstrates that entrectinib, while generally well tolerated, is rarely associated with early-onset myocarditis with acute heart failure. Our patient demonstrated myocarditis within three days of initiation of entrectinib, earlier than in the only other case reported. Clinicians should remain vigilant for early signs of myocarditis in patients on entrectinib and be aware that the triangular QRS-ST-T waveform ECG pattern, while usually indicating occlusive myocardial infarction, can occur in myocarditis. Knowledge of this association may prevent misdiagnosis and inappropriate management in patients presenting with this high-risk ECG pattern.
